# Final Consumer Options to Control and Prevent Foodborne Norovirus Infections

**DOI:** 10.3390/v11040333

**Published:** 2019-04-09

**Authors:** Susana Guix, Rosa M. Pintó, Albert Bosch

**Affiliations:** 1 Enteric Virus Laboratory, Department of Genetics, Microbiology and Statistics, University of Barcelona, 08028 Barcelona, Spain; rpinto@ub.edu (R.M.P.); abosch@ub.edu (A.B.); 2 Institute of Nutrition and Food Safety (INSA·UB), University of Barcelona, 08291 Santa Coloma de Gramenet, Spain

**Keywords:** noroviruses, foodborne infections, consumers, prevention, control

## Abstract

Norovirus (NoV) causes about one-fifth of all cases of foodborne diseases and is a foremost cause of domestically acquired foodborne acute gastroenteritis and outbreaks. NoV infections are often associated with the consumption of contaminated fresh and ready-to-eat produce, fresh and frozen berries, raw/undercooked bivalve mollusks and products which become contaminated during handling. Despite many industrial efforts to control and prevent NoV contamination of foods, the prevalence of NoV in high-risk foodstuffs at retail is still significant. Although certain consumer behaviors may even increase the risk of virus transmission, interventions aiming at changing/implementing consumer habits may be considered as opportunities for risk mitigation. This review aims at providing an update on the progress made in characterizing the effect that consumer habits, which are most critical to prevent NoV transmission (food choice and hygiene, disinfection and cooking during food preparation), may have on reducing the risk of NoV infection. A better understanding of the options for NoV control and prevention may be translated into innovative educational, social or even technological tools targeting consumers with the objective of mitigating the risk of NoV transmission.

## 1. Introduction

The increase in norovirus (NoV) research over the last 15 years has been tremendous and has provided relevant improvements and widespread availability of diagnostic/detection methods. We now know that NoV cases in humans are ubiquitous and affect all age groups. Among the seven current genogroups (G) that have been described for NoVs, GI, GII and GIV can infect humans [1]. Of the over 40 existing genotypes, a few of them belonging to GII (GII.4, GII.17, and GII.2) account for the vast majority of NoV cases worldwide. Although NoV gastroenteritis usually lasts only a few days, more severe disease and cases of chronic gastroenteritis may also occur among the elderly and immunosuppressed [2].

NoV disease estimates associate NoV with 18% of the total foodborne diseases burden worldwide, 680 million cases in 2010 [3,4]. In Europe, the WHO states that NoV causes 15 million cases (and 400 deaths) annually, although the number of NoV deaths globally are estimated to be 212,000 every year, with approximately 99% occurring in middle- and high-mortality countries [3]. Estimates of NoV disease incidence in industrialized countries range from 380 to over 1000 cases per 10,000 population, and the number of infections is believed to be underestimated due to underreporting [5]. Since NoV monitorization and outbreak investigations are not harmonized between different countries, the proportion of NoV cases associated with foodborne and waterborne transmission varies depending on the study, although it is estimated to be 17% (95% IC 16–47) [6].

The burden of NoV outbreaks is especially significant in restaurants and hotels, as well as in semi-closed institutions, where secondary transmissions often occur, and as many other foodborne pathogens, domestic/kitchen outbreaks are also common. According to data from the European Centers for Disease Control (ECDC), NoV was the etiological agent in 7, 8, and 4% of all reported foodborne outbreaks, in 2015, 2016 and 2017, respectively, following *Salmonella*, bacterial toxins and *Campylobacter*, with a strong association with settings where food was prepared and/or served by catering services, restaurants and household environments [7,8,9]. Bivalve mollusks were the contaminated foodstuff in 27.8% of all NoV outbreaks, and fruits and vegetables caused 11% of them [8]. The largest foodborne NoV outbreak occurred in Germany in 2012, with almost 11,000 young individuals affected after consuming frozen strawberries imported from China [10]. The largest waterborne NoV outbreak occurred in Spain in 2016, and it was linked to contaminated drinking spring water from office water coolers, bottled at source in Andorra [11]. It caused over 4000 cases in individuals and highlighted that the microbiological quality of water based on bacterial parameters is an inappropriate proxy to evaluate the risk of viral infections.

NoV may contaminate a wide variety of food products at pre-harvest or post-harvest stages. Among those foods at risk of pre-harvest contamination, usually through contact with fecally contaminated water, bivalve mollusks, soft fruits, and ready-to-eat (RTE) leaf vegetables and produce are most commonly associated with foodborne outbreaks. As a consequence of food trade globalization, many outbreaks are caused by products imported from third-world countries. Post-harvest contamination most likely results from poor hygiene practices during food handling, and hence uncooked or lightly cooked products which are highly manipulated are the foods with a higher risk. Surfaces employed for food preparation, as well as other types of fomites, may also act as vehicles for foodborne virus transmission.

To date, none of the food safety and process hygiene microbiologic criteria reflected in European regulations include virological determinations, but the documents related to the occurrence and control strategies on foodborne viruses published by the European Food Safety Authority (EFSA) during the past 5 years highlights a profound concern on the subject [12,13,14,15]. Indeed, there is a widespread belief within the food sector that a regulatory standard regarding the control of NoV, as well as hepatitis A virus (HAV), the other virus most commonly found as a food contaminant, in selected foodstuffs (bivalve mollusks or fresh produce) will be established soon once enough quantitative data on viral load has been gathered to allow the determination of maximum acceptable levels of contamination.

NoV alerts have been common lately in the Rapid Alert System for Food and Feed (RASFF) system (https://ec.europa.eu/food/safety/rasff/portal_en), an online system which provides food and feed European control authorities with an effective tool to exchange information about measures taken responding to serious risks detected in relation to food or feed. Alerts from 2017 described NoV in 29% and 28% of fruits and vegetables (mostly frozen berries), and bivalve mollusks (mostly raw oysters and other frozen bivalves), respectively [16].

The main measures of control and prevention to reduce the likelihood that consumers buy food that is contaminated with NoV must be reinforced by industrial actors throughout the entire food chain, especially in the shellfish sector, in agriculture and in the catering and restoration sector [12,13,17]. Shellfish producers must ensure the quality of the water in the growing areas. Several authors have confirmed the higher levels of NoV contamination in areas close to large populations, especially those lacking wastewater treatment systems [18] or having old lagoon-based sewage treatment plants [19]. In a study performed in the UK, water temperature < 5 °C and larger volumes of sewage discharges were identified as risk factors for NoV contamination in shellfish [20].

Likewise, in primary production in agriculture, it is important to control the quality of irrigation water, wash water, the origin and quality of fertilizers and natural fertilizers, and the hygiene of the facilities. The main risk factors in this sector include environmental factors, such as heavy rainfall or floods that increase the transfer of sewage or sewage effluents to irrigation water sources or fields, the use of sewage-contaminated water for irrigation or pesticide treatment, and contamination by food handlers or equipment during harvest or on farm post-harvest steps [14,21].

In the catering and restoration sector, as well as in retail, correct and rigorous hygiene by food workers is also crucial in order to prevent the transmission of any virus to food or its spread through the facilities and surfaces. The main risk factors for NoV contamination are washing with contaminated water (if availability of potable water is compromised), and handling by an infected person. Hygiene practices during distribution, at retail and in caterers in Europe are very diverse. There is no specific regulation on washing/disinfection procedures in vegetables, use of gloves, etc., other than cleaning with potable water and the requirement to achieve the microbiological criteria. In the case of food handlers with gastroenteritis, the Codex Alimentarius Commission’s guidelines recommended to return to work only after having passed a period without symptoms of diarrhea and vomiting [22], but no specific regulation exists. Environmental or equipment contamination that may pose a risk for later cross-contamination events would only be significant in scenarios with infected people in the establishment. Of note, the number of NoV asymptomatic infections can be considerable, so it is important to maximize hand and surface hygiene practices at all times.

Despite all industrial efforts, the prevalence of NoV in foodstuffs at retail may still be significant, especially in high risk foods, and final consumers may also contribute to reduce exposure to NoV, spare many infections annually, and reduce costs related to health care and productivity losses due to sickness leave from work. Among many critical points, those which may be important from retail to fork to reduce NoV risk include food choice, transportation and storage, and preparation (hygiene, washing and cooking). Specific knowledge of NoV prevention and control by regular consumers has recently been evaluated in the US, and revealed that 85% of respondents were familiar with the term “norovirus”, but had a greater awareness of foodborne bacteria than foodborne viruses such as NoV. In addition, the survey revealed a lack of understanding that the primary mode of transmission for NoV infection is fecal to oral and the misperception that meat and poultry are sources of NoV infection were highlighted [23].

**Figure 1:**
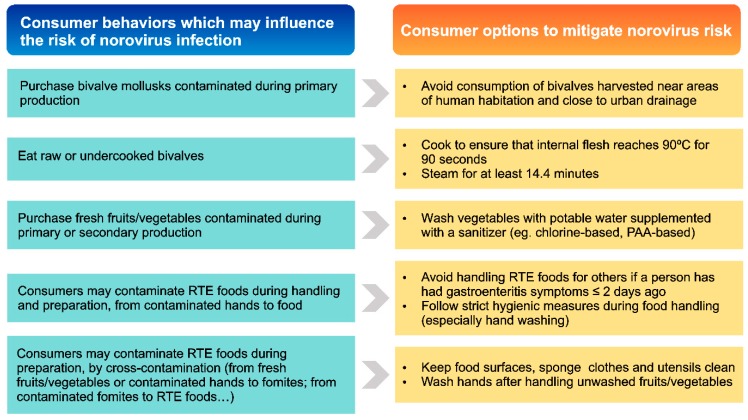
Major consumer habits and behaviors which may increase norovirus risk of infection, and options available for consumers to mitigate the risk.

Consumer lack of knowledge on food safety measures contributes to the occurrence of NoV infections, but consumer education and acquisition of specific skills and behaviors may also offer opportunities for intervention to mitigate the risk. In other words, consumer behavior is both a core problem and part of the solution. This review aims at providing an update on the progress made in some of the most important consumer habits and behaviors which may have an effect on NoV risk of infection (Figure 1). 

## 2. Food Choice: Is It Possible to Prevent NoV by Selecting Products with Low Risk?

Despite the publication of a standardized methodology, in 2013, to qualitatively and quantitatively detect NoV genomes in selected food matrices [24], the number of studies describing the prevalence of NoV in different high risk foods (bivalve mollusks and fresh produce) is still limited. In addition, most studies do not report information on the limit of detection and quantification, and this makes it difficult to compare data from different laboratories in order to be certain that the level of NoV contamination correlates with the geographic origin and/or time of the year. Additionally, it is worth emphasizing that molecular techniques do not provide information on viral infectivity, and that the ratio of genome:infectious viruses may be different in each particular environmental sample, depending on many factors such as time from shedding, climate conditions, etc. Data on NoV infectivity in correlation with genome titers will be indispensable to assess the risk to human health and establish maximum levels of acceptable genome contamination. A study performed in the UK determined that the geometric mean of NoV genome levels in oysters involved in outbreaks was of 1.0 × 10^3^ genome copies/g, while it was only 1.2 × 10^2^ for non-outbreak-related oysters [25]. A more recent study in the US has described much lower levels in oysters associated with outbreaks, ranging from 1.5–82 genome copies/g [26].

Data on NoV genome prevalence on bivalve mollusks are reported in Table 1. Most studies are not quantitative, and positivity rates for NoV in different species of mollusks range from 25–76% in European countries such as Belgium, Italy, Montenegro, Netherlands, Spain, UK, and Ireland, and slightly lower in France (9–22%). Positivity rates were higher for GII than for GI in >70% of all revised studies, with average rates of 20% (range 2–67%) for GI and 29% (range 5–56%) for GII. For European oysters, the average positivity rate is 52% (range 9–76%), without apparent differences in GI and GII prevalence, both being of approximately 35%. In other parts of the world, the number of studies is rather low. Prevalences are relatively low in Japan, China, Australia and the US and significantly higher in Vietnam or Morocco.

On average, the level of contamination ranges between 10^2^–10^4^ genome copies/g of digestive tissue, although levels as high as 10^9^ have also been occasionally reported. Seasonality has been described for shellfish contamination in France, Ireland, Montenegro and the UK [13,27,28,29,30,31], although this is not a universal phenomenon, and data from countries like Italy or Spain are conflicting [32,33,34,35].

Despite certain differences depending on the geographic origin or time of the year, it is not possible for consumers to control the risk of NoV infection by selecting low-risk bivalve products. However, educational programs could be developed to make consumers more alert to the risk of viral contamination in bivalve mollusks, especially when harvested near areas of human habitation and close to urban drainage.

Regarding fruits and vegetables, most recent prevalence studies performed in Europe on leafy greens, mostly lettuce, report relatively low prevalences, ranging from 0–2% [36,37,38,39] (Table 2). However, older studies and studies from other countries also report notably high NoV prevalences, ranging from 12–54% in leafy greens, including RTE samples, and 6–34% in berries [40,41,42]. Indeed, both in Europe and in the US, berries caused more than half of NoV outbreaks linked to vegetables between 2004–2012 [43]. Data on viral load are extremely scarce, with numbers in the range of 1.4 to 9 × 10^6^ genome copies/25 g.

Indeed, primary production conditions of fresh produce may vary considerably between geographical locations and countries, and circumstances such as agricultural inputs and technologies, water availability and quality, hygienic practices, or epidemiological environments may affect the occurrence and level of virus contamination that will finally get to consumers. Special attention must be paid to berry mixes, which are often imported frozen and which may contain ingredients from different origins, adding complexity to traceability and probability of contamination. Finally, whether risk of NoV contamination could be different in organic produce has not been consistently addressed yet.

## 3. Survival of NoV on Fruits and Vegetables and Elimination during Washing and Sanitation

The lack of in vitro assays which could be used on a routine basis to measure NoV infectivity has hampered the execution of survival studies, and most data, if not all, come only from measuring survival of genome detection or the use of viral surrogates, such as Murine Norovirus (MNV) and Feline Calicivirus (FCV). Unfortunately, the human intestinal enteroid model developed for NoV replication in vitro [62] has not yet been routinely used to perform survival studies and validate all these data in terms of infectivity.

An extensive review, including survival and elimination of NoV genomes on food, has been published by Cook et al. [63]. At refrigeration temperatures, the time to reduce the initial NoV load by 1 log_10_ was higher than 2 weeks on lettuce, and even higher than 58 days on tomato ketchup. At room temperature, it varied between 9–17 days on lettuce. Although 1 log_10_ reduction was observed in 3 days on strawberries, survival was much longer on raspberries under the same conditions. The robustness of NoV to freezing has also been demonstrated with reductions lower than 1 log_10_ in all experiments.

Washing produce in potable water is a common practice at the domestic level, but addition of a sanitizer is not a widespread practice. Among sanitizers approved in the European Union (EU) and United States (US), chlorine bleach products are the ones most readily available for domestic use. Sensitivity of NoV to chlorine has also been evaluated using the human intestinal enteroid model [64]. Although log_10_ reductions were not estimated, the experiments confirmed that total chlorine concentrations >50 ppm for 1 min inactivated all tested NoV strains compared to untreated controls. For concentrations between 50–600 ppm, infectious viruses were no longer detected, while viral genomes were still amplifiable by RTqPCR. Some official agencies from EU member states recommend consumers to wash fruits and produce that are going to be eaten raw and without peeling by soaking them for 5 min in potable water with 1 teaspoon of bleach (4.5 mL) per 3 L of water, and rinsing them with plenty of running water after that. The bleach must be labeled as “suitable for disinfection of drinking water”, and several chrorine-based products are commercially available for final consumers in most countries. Unfortunately, data on the actual implementation of this measure are not available.

Chlorine is also the first choice in the fresh-cut industry, although the possible formation of carcinogenic chlorinated compounds like chloramines or trihalomethanes in water has called into question the general use of chlorine in food processing. Indeed, a few countries, such as the Netherlands, have forbidden its use due to environmental concerns. Note that other types of sanitizers, such as peracetic acid (PAA), ozone and chlorine dioxide, can be used in the fresh-cut industry, and novel sanitizers such as levulinic acid (LVA) and sodium dodecyl sulfate (SDS) or food-derived substances (such as heat-denatured lysozyme) are also being evaluated for the post-harvest washing of berries, but their use in private households would be difficult [65,66]. Interest in PAA as a disinfectant in the food processing and water industry has increased in the recent years due to its effectiveness at room temperature, lower toxicity, lower susceptibility to its effect being affected by pH or the presence of organic matter, a long shelf life and reasonable cost [67].

The effectiveness of several sanitizers on NoV has been addressed by several laboratories always by measuring reduction on viral genomes or using the MNV surrogate (Table 3). While washing with water alone achieves 0.1–1.8 log_10_ reductions in viral load, addition of a sanitizer increased the effect by 0.5–2.6 log_10_ reductions. Thus, the use of sanitizers for washing soft fruits and produce would reduce the risk for NoV infection by almost 1000 fold; in other words, risk could be completely eliminated in samples containing less than 10^2^–10^3^ viruses per serving. However, since quantitative prevalence data on such food products are still insufficient, it cannot be determined whether such disinfection procedures would be sufficient to mitigate the risk in most scenarios. In addition, as opposed to washing with water alone, the use of sanitizers also reduces the occurrence of viruses in the residual wash water, lowering the likelihood of cross-contamination in the environment.

## 4. Safe Cooking Methods for Bivalve Mollusks to Ensure NoV Inactivation

Published data on human NoV thermal inactivation on shellfish samples since publication of the last comprehensive review on the subject [72] are very limited. A treatment ensuring that the internal temperature of the mollusk flesh is maintained at 90 °C for a minimum of 90 s is recommended as a safe virucidal treatment [22], and is applied by current EU rules to a broad range of bivalve mollusks from Class B and C production areas in the EU and third-world countries importing into the EU. However, infographic guidelines from the US Centers for Disease Control (CDC) to prevent NoV outbreaks (https://www.cdc.gov/vitalsigns/norovirus/infographic.html#infographic) recommend consumers to cook shellfish over 63 °C, following the FDA’s food code [73].

Despite the lack of microbiological criteria for NoV in the EU, NoV determination is performed at border inspection and positive results are alerted in the RASFF system. Of note, despite the assumption that cooked bivalves may be considered safe, many alerts in the RASFF system correspond to frozen cooked clams. So far, epidemiological data indicate that most shellfish outbreaks are associated with raw or undercooked bivalves sold fresh or even frozen [74,75] but, in 2018, an outbreak following the consumption of commercially heat-treated bivalve mussels occurring in Spain was reported to the RASFF system (2018.1014), suggesting that either the industrial thermal processing was defective or that it was not virucidal enough.

Even though most consumers may have the idea that eating raw or insufficiently cooked bivalves poses a health risk, the culinary practices are enormously diverse, and current temperatures achieved by most cooking practices in private homes (immersion in boiling water, light steaming until the shells open, frying, microwaving, or oven baking) are largely unknown. In addition, thorough cooking may change the organoleptic characteristics of most mollusks. In mussels, for instance, the mean internal temperature of 90 °C for 90 s may be reached after boiling for 170 s but may not be reached after 300 s of steaming; steaming for 180 s may reach an internal temperature of 63 °C [76], but extrapolating these data to all bivalves may be incorrect. While boiling for 170 s achieves >3.5 log_10_ reduction for infectious HAV, for NoV the treatment induces a reduction of 2.3 log_10_ in genome titers (1.9 log_10_ for HAV genomes). Steaming for 180 s induces a reduction of only 1.5 and 0.3 log_10_ for infectious HAV and NoV genomes, respectively. Reported D-values or decimal reduction times, described as the time required at a given temperature to achieve a log reduction, are comparatively shown in Table 4 for HAV and NoV in mussels as measured by the reduction in infectious viruses and viral genomes. According to a scientific opinion published by EFSA in 2015 based on a thermal inactivation model constructed using HAV data, treating at 63 °C for 14.4 min would achieve equivalent viral reductions to 90 °C for 90 s [15], but this would still be uncertain for NoV. These data show that recommendations indicating cooking procedures at 63 °C (steaming for example) should be performed for at least 14.4 min to ensure a significant >1000-fold risk reduction.

## 5. Spread of NoV by Infected Food Handlers and Lack of Personal Hygiene

Food handlers have been implicated in the transmission of NoV during food production, including harvest, processing, packaging, distribution, at the point of retail sale, and also at home [81]. The occurrence of gastroenteritis symptoms and poor hand hygiene increases the likelihood of the virus being deposited or transferred from hands to food or food preparation areas.

The role of food handlers in private households has not been investigated thoroughly, but it is obvious that it also occurs. Although the likelihood of shedding the virus into the environment is reduced after the symptomatic phase, shedding can still occur on average up to two weeks after symptoms have stopped, and for much longer in immunocompromised individuals. In order to reduce this risk, professional food handlers should remain off work until they are non-infectious, which has been suggested as 48 h after symptoms have ceased [82], and a recommendation not to prepare food for others when sick or at least 2 days after symptoms stop was posted by the CDC. A recent quantitative risk assessment study revealed that if employees were not excluded when ill, a 226% increase in the number of infected customers should be expected, and full compliance would decrease the numbers to 75% [83]. Although significant, these numbers still indicate that the reinforcement of hygiene practices among workers continues to be required to reduce the risk of virus transmissions. In addition, fecal excretion of NoV by asymptomatically-infected individuals is also common and may also play a role in spreading infections during food preparation.

The occurrence of symptomatic and asymptomatic infections in a household, the compliance and efficacy of hand hygiene, and cleaning and disinfecting fomites to prevent cross-contamination events are the most critical factors that general consumers should take into account to prevent foodborne NoV infections at home.

### 5.1. NoV Asymptomatic Carriers

The prevalence of asymptomatic carrier state among adults reported by different authors ranges between 1–16% [2,84,85]. Some of these studies have been performed on populations of asymptomatic food handlers unrelated to outbreaks and have found similar positivity rates [86,87]. A study performed in Indonesia described that 7 out of 18 (39%) healthy adults prospectively screened for one year shed NoV at some point during the study period [88]. Asymptomatic shedding in children has been reported to be <1% in preschool Swedish children [89], 11.7% in children under 5 years old in Nicaragua [90], or even >30% in England [85], Mexico [91], Cameroon [92], or South Africa [93], with prevalences which were even equivalent to those observed among children with acute gastroenteritis in this latter study.

Regarding levels of viral excretion, challenge studies with healthy adults have shown that individuals with gastroenteritis show concentrations of virus shedding that are approximately 10-fold higher compared to those who do not show symptoms and that the total amount of particles shed during 2 weeks by individuals who have had gastroenteritis is also approximately 10-fold higher [94,95], although other studies on subjects related to outbreak investigations have not found statistically significant differences in levels and duration of shedding between symptomatic and asymptomatic subjects [96,97]. In children, some semi-quantitative analyses of viral load in symptomatic and asymptomatic subjects have also revealed a significant higher viral load in specimens from symptomatic children [93].

The risk factors for symptomatic NoV infections identified in adults and children >5 years old from industrialized countries include previous contact with individuals with infectious intestinal disease symptoms, living in a household with a baby in nappies, foreign travel, eating shellfish [98] and treatment with proton pump inhibitors [99]. In preschool children, infections have been associated with attending day care centers [100] and previous antibiotic use [101]. No risk factors have been clearly identified for asymptomatic norovirus infections. Finally, although still recent, it is worth mentioning that a link between the secretor status which is determined genetically, gut glycobiology, intestinal microbiota composition and susceptibility to NoV infection has begun to be elucidated [102]. Whether probiotics and/or dietary interventions could be used as treatment or preventive strategies is still an open research area, with few published successful examples [103,104].

### 5.2. Hand Hygiene and Disinfection

Hands contaminated with NoV play a key role in its transmission, and disinfection is an essential measure for interrupting it. However, due to the absence of a practicable cell culture system for human NoV, evaluating the health significance of NoV genomes occurring on hands as well as the efficacy of disinfectants is also difficult.

In a study performed during a challenge trial, 25.4% (18/71) of the hand rinse samples collected from six infected volunteers were positive for NoV, with an average of 7.2 × 10^3^ genomic copies per hand [105]. Another recent study performed among patients and healthcare workers at long-term care facilities reported NoV contamination in 73.3% of residents’ hands and in 20% of healthcare workers’ hands. The viral load on the hands of symptomatic residents was 1.6 × 10^6^ genome copies (range, 2.5 × 10^2^–7.9 × 10^7^), 7.9 × 10^4^ genome copies (range, 4.8–5.8) on hands of postsymptomatic residents, and 5.0 × 10^3^ genome copies (range, 2.5 × 10^2^–7.9 × 10^4^) on hands of postsymptomatic workers [106]. Considering the concentration of NoV that may be present in the stool of infected individuals, this could correspond to as little as 0.01 mg of contaminating fecal material.

NoV was detected in approximately 5% of hand rinse samples from randomly selected mothers with children under the age of 5 years old in Tanzania, without a correlation with fecal indicator bacteria levels [107]. The average concentration in mothers’ hands was 6.3 × 10^1^ and 1.6 × 10^3^ genome copies per two hands in the rainy and dry season, respectively. 

Most studies aiming at investigating the virucidal activity of alcohol-based hand sanitizers and/or regular hand washing have used NoV surrogates such as MNV and FCV, so their efficacy against human NoV still remains inconclusive nowadays. Since there is concern on extrapolating data on MNV and FCV to human NoV, it still remains unclear if human NoV would behave similarly to most nonenveloped viruses. Contrary to what has been observed for enveloped viruses such as influenza, the reduction obtained for the majority of non-enveloped viruses by ethanol-based formulations would only be partial (<4 log_10_ of infectious viruses) [108]. MNV is usually inactivated by ethanol between 70% and 90%, but for some other viruses such as HAV, poliovirus, polyomavirus, foot-and-mouth disease virus and also FCV, ethanol 80% would be insufficient. Compared with the propanols, ethanol has shown a superior efficacy against non-enveloped viruses, including MNV and FCV. The addition of acids to the formulation such as phosphoric acid, citric acid, peracetic acid or urea has been shown to substantially improve the virucidal efficacy of ethanol for most studied viruses [108,109].

Published data using measurements of human NoV genome detection indicate that ethanol is relatively ineffective for disinfecting finger pads, giving at most a 0.5 log_10_ reduction in genome copies [110]. Water rinse only removes 0.5–1.5 log_10_ and hand washing would be the most effective method removing 1–2 log_10_ [110]. However, other studies have shown removal results higher than 2.6–3.3 log_10_ in NoV genome copies after hand washing with soap and water [111,112], highlighting the need to perform additional studies.

In conclusion, alcohol-based hand sanitizers are advisable, but since their efficacy may not be optimal, especially for the epidemiologically relevant human NoV in scenarios including infected subjects with symptoms, they should be used in addition and not as a substitutive to hand washing with soap and water.

### 5.3. Environmental and Fomite Contamination

Fomites have been shown to have an important role in seeding outbreak events. Much of this evidence comes from the healthcare and community sectors, but little is known on their role in household transmission events. Contamination of household fomites that may eventually contaminate food would mostly come from contamination with fecal material from infected individuals, although contaminated produce as a source of contamination cannot be completely ruled out. Although elements such as kitchen surfaces, fridge handles or cooking utensils would be the most critical ones, other less investigated elements such as sponge clothes, remote switches or mobile phones may also play a role in transmission. Indeed, mobile phones have been identified as a potential reservoir for nosocomial enteric viruses in healthcare environments, as has been observed for bacteria. A study performed in France detected the presence of virus RNA in 38.5% of screened mobile phones, with a significant stronger association with the phones of pediatric healthcare workers [113].

Data from kitchen environmental contamination are only available for catering companies or healthcare institutions. A one-year survey study performed in catering companies in the Netherlands identified a positivity rate of environmental swabs taken from surfaces in the kitchens of 0.96% for companies without recently reported outbreaks and 29% for establishments with recently reported outbreaks. Positive samples included refrigerator grips, mixing or cutting machines, the grip of a bread knife, a salt-and-pepper set and a soap dispenser [114]. A few years later, a large survey during NoV peak season detected NoV RNA in 3.4% of surface preparation areas of elderly homes, 0.9–7% in kitchens from different hospitals and 1.6% in kitchens from catering companies [115]. During outbreak investigations in Japan, 0.7% of the swab specimens of kitchens were positive for NoV [116]. As reported by a study performed in Finland after screening restaurants and canteens during outbreak investigations, NoV environmental contamination is not associated with lower hygiene levels when based on visual inspection [117]. It is difficult to anticipate from these data whether the prevalence of environmental contamination in private kitchens would be different from these ones.

Unfortunately, none of these studies have so far included a quantitative analysis of NoV levels of contamination, or confirmed that detected genomes maintain infection ability, making it difficult to evaluate the risk of transmission to individuals. A study screening several office fomites found the highest average concentration of NoV genomes on the light switches (1.4 × 10^2^ copies/100 cm^2^) [118].

As other food associated viruses, NoVs have been shown to persist in the environment even when exposed to harsh conditions for long durations of time. NoV concentrations on different surfaces (stainless steel, ceramic and formica) has been shown to decline only between 1.5–2.3 log_10_ genome copies in 42 days, depending on the tested surface [63,119]. Again, since these persistence studies can only be performed by molecular RTqPCR assays, it is impossible to confirm that the RNA detected remains infectious. The use of the human intestinal enteroid model [64] or alternative molecular methods such as the use of viability RTqPCR assays [120,121] for survival studies is required to confirm whether surviving genomes remain infectious for such long periods of time.

### 5.4. Food Contamination by Contact with Fomites and Handling

The transfer of enteric viruses by contact with fomites (food preparation surfaces, utensils, sponge clothes…) and by handling during food preparation is highly likely, but has been quantitatively investigated on few occasions. Escudero et al. [119] identified the transfer of NoV and surrogate viruses from artificially contaminated stainless steel, ceramic and formica onto lettuce and turkey in artificial contamination experiments. The transfer efficiency ranged from 0 to 26% for lettuce and from 55 to 95% for sliced turkey deli meat. Stals et al. [122] identified lower transference efficiencies, of 5% and 8% from gloves to ham and lettuce, respectively, although the results of this study were obtained by swabbing food surfaces, rather than testing the whole food sample. They also reported a transference efficiency of 38% from different surfaces to gloves. Rönnqvist et al. [123] observed that NoV may be easily transferred from contaminated hands to latex gloves during gloving, and reported transferability percentages of 1.2–1.5% from latex gloves to cucumbers during simulated sandwich preparation. Still using the MNV model, Grove et al. [112] reported greater transferability from a contaminated stainless steel spigot to a clean hand (24%) compared to transferability from hand to spigot (0.6%). During the chopping of Romaine lettuce, MNV was transferred from either a contaminated cutting board (25%) or knife (~100%) to lettuce at a significantly greater rate than from contaminated lettuce to the board (2.1%) and knife (1.2%). Finally, an average of 1.1% was transferred from contaminated hands to lettuce, and vice versa during handling.

Transfer efficiency may depend on many factors, including the pressure applied, contact time, the texture of a contact surface and especially whether the contact surface is wet or dry. Thus, Sharps et al. [124] identified wet inoculation of a virus cocktail including NoV and MNV transferred at a rate of 20–70% from gloved fingertips onto stainless steel, blueberries and grapes, while dry inoculation transferred at a much lower rate of 4–12%. Similarly, Tuladhar et al. [125] also found that, when dried, MNV transferability from finger pads to stainless steel was reduced from 13 ± 16% down to 0.1 ± 0.2%. MNV was also transferred at 7 ± 8% to cucumber slices and 0.3 ± 0.5% to tomatoes after 10 min of drying.

## 6. Conclusions

While food safety authorities play a crucial role in developing and implementing strategies towards mitigation of the risk of foodborne infections, as well as in promoting safe consumer behaviors, final consumers are also partly responsible for their health and their family’s health, and may also be a key factor for improving control and prevention measures. Importantly, from retail to consumption, certain consumer habits contribute to increase the risk of infection, but they also offer intervention opportunities to reach a significant risk reduction and safer food, being at the same time a core problem and a solution. A comprehensive and science-based understanding of the most critical points and knowledge gaps may lay the foundations for the development of innovative educational, social or even technological tools addressed to consumers aimed at reducing the risk of NoV transmission.

## Figures and Tables

**Table 1:** Prevalence studies of norovirus (NoV) in bivalve mollusks.

Country	Type of Shellfish	Production Areas	Dates of Sampling	NoV Prevalence (Total Number of Analyzed Samples)	Prevalence of NoV GI	Prevalence of NoV GII	Data on Viral Load ^a^ → % Positive Samples	Ref.
**EUROPE**								
Belgium	Oysters, clams and mussels (raw and frozen)	Seafood company	2012–2013	32.3% (*n* = 65)	24.6%	13.8%	3.3 × 10^3^–1.4 × 10^4^ →42.6%	[44]
France	Oysters	ND	ND	14% (*n* = 78)	ND	ND	ND	[13]
France	Mussels	Thermally-treated shellfish; imported from different countries as frozen	2008	21.7% (*n* = 83)	8.4%	11.4%	ND	[42]
France	Oysters	A and B	2010–2011	9% (*n* = 387)	1.6%	8.3%	Range: 9.3 × 10^1^–2.2 × 10^2^	[27]
Ireland	Oysters	A and B	2005–2007	37.1% (*n* = 167)	ND	ND	ND	[45]
Ireland	Oysters	A and B	2009–2011	88.1% (Nov-March) 50.9% (April-Oct) (*n* = 113)	ND	ND	Average Nov-March: 1.3 × 10^3^ Average April-Oct: 2.1 × 10^2^	[13]
Ireland	Oysters	Point of sale	2015–2016	84% (*n* = 25)	ND	ND	Geometric mean (95% CI): 69 (40–120)	[30]
Italy	Clams and mussels (before and after depuration)	A and B	2005–2006	8.3% (0% in depurated samples) (*n* = 120)	3.3%	5%	ND	[32]
Italy	Clams, mussels and oysters	B	2008–2009	51.4% (*n* = 70)	37.1%	48.6%	ND	[46]
Italy	Clams, mussels, oysters and others	A and B	2008–2012	51.5% (*n* = 336)	26.5%	45.4%	Average A area: 3.1 × 10^2^ Average B area: 1.9 × 10^3^	[47]
Italy	Mussels and razor shells	A and natural beds	2011–2012	18.7% (*n* = 59)	6.8%	11.9%	ND	[48]
Italy	Clams, mussels, oysters and others	A and product at retail	2003–2011	4.2% (*n* = 4359)	ND	ND	ND	[49]
Italy	Mussels and clams	A and B, as well as shellfish from registered and unregistered retailers (street vendors)	2007–2010	57.5% (*n* = 163)	29.4%	56.4%	ND	[33]
Italy	Mussels	A and B	2014–2015	23.1% (*n* = 108)	9.3%	20.4%	Average GI: 1.0 × 10^2^ Average GII: 0.6 × 10^2^ (range 1.2 × 10^2^–5.8 × 10^7^)	[50]
Italy	Mussels, clams, oysters, and others	A and B	2013–2015	14.2% (*n* = 253)	1.6%	12.2%	ND	[51]
Montenegro	Mussels	A and B	2015–2016	43% (*n* = 72)	19.4%	37.5%	Range GI: 10–1.2 × 10^3^ Range GII: 10–1.2 × 10^4^	[31]
Netherlands	Oysters	Point of sale	2015–2016	31% (*n* = 29)	ND	ND	Geometric mean (95% CI): 49 (40–91)	[30]
Spain (Galicia)	Clams, cockles, mussels	B and C	2005	56% (*n* = 41)	7.3%	53.7%	Range: 5.6 × 10^1^–1.5 × 10^4^	[52]
Spain (Galicia)	Mussels	B and C	2010–2012	49.4% (*n* = 81)	8.6%	30.8%	Range: 5.9 × 10^3^–1.6 × 10^9^	[34]
Spain (Galicia)	Clams, cockles, mussels	B	2011–2012	45.2% (*n* = 168)	32.1%	25.6%	<10^2^→9.5 % 10^2^–10^3^→31.6 % 10^3^–10^4^→37.9 % 10^4^–10^5^→20 % >10^5^→1 %	[35]
UK	Oysters	B	2004–2006	48% (*n* = 14)	41.4%	40%	ND	[28]
UK and Scotland	Oysters	39 areas, A, B and C	2009–2011	76.2% (*n* = 844)	67.4%	54.5%	<10^2^→63.5 % 10^2^–10^3^→21.9 % 10^3^–10^4^→13.5 %	[29]
UK	Oysters	Point of sale	2015–2016	71.7% (*n* = 434)	ND	ND	Geometric mean (95% CI): 78 (40–277)	[30]
**ASIA AND AUSTRALIA**								
Australia	Oysters	Growing areas with risk of pollution	2010–2011	1.7% (*n* = 120)	0%	1.7%	ND	[53]
Australia	Oysters	All major oyster harvest areas within the country	2014–2015	<2% (*n* = 300)	0%	0%	ND	[54]
China	Clams, mussels, oysters and others	Retail	2009–2011	13.3% (*n* = 840)	ND	ND	<10^2^→37.4% 10^2^–10^3^→46.1% 10^3^–10^4^→13.9% >10^4^→2.6%	[55]
Japan	Oysters	A and B	2002	9% (*n* = 191)	7.4%	1.6%	<10^2^→35.3 % 10^2^–10^3^→41.2 % 10^3^–10^4^→17.6 % >10^4^→5.9 %	[56]
Vietnam	Oysters	ND	2016–2016	ND (*n* = 34)	79%	42%	Max GI: 2.4 × 10^5^ Mas GII: 2.3 × 10^4^	[18]
**AFRICA**								
Morocco	Clams, cockles and oysters	ND	2006–2010	29.9% (*n* = 77)	20.8%	18.2%	Average clams: 2.1 × 10^2^ Average cockles: 2.8 × 10^2^	[57]
**UNITED STATES (US)**								
US (Louisiana)	Oysters	A and B	2013	0.2% (*n* = 440)	0%	0.2%	3.4 × 10^3^	[58]
US	Oysters	ND	2007	3.9% (*n* = 338)	1%	2.9%	ND	[59]

^a^ Viral load is expressed as genome copies per gram of digestive tissue; ND: Not determined; CI: confidence interval.

**Table 2:** Prevalence studies of norovirus (NoV) in fruits and vegetables.

Country of Commercialization	Type of Vegetables	Country of Production	Sampling Period	NoV Prevalence (Total Number of Analyzed Samples)	Data on Viral Load ^a^	Ref.
**EUROPE**						
Belgium, France	Leafy greens	Belgium, Canada, France, US, Mexico, Spain, Poland/Serbia	2009–2010	Belgium: 33.3% (*n* = 6) France: 50% (*n* = 6)	ND	[40]
Belgium, France	Soft red fruits	Belgium, Canada, France, US, Mexico, Spain, Poland/Serbia	2009–2010	Belgium: 34.5% (*n* = 29) France: 6.7% (*n* = 150)	ND	[40]
France	Berries	Serbia, Chile, Bulgaria, Poland, Spain, Morocco, Turkey	ND	16% (*n* = 200)	ND	[42]
France	Lettuce	Spain, Italy, Belgium, France and Tunisia	ND	12.4% (*n* = 210)	ND	[42]
Greece, Serbia and Poland	Fresh lettuce	Greece, Serbia and Poland	ND	1.3% GI (*n* = 149) 0.8% GII (*n* = 126)	6–23	[38]
Italy	Fresh and RTE green vegetables	ND	2011–2012	Fresh leafy veg: 0.1% (*n* = 1372) RTE veg: 0% (*n* = 1160)	ND	[36]
Italy	RTE vegetables	ND	2014–2015	0% (*n* = 911)	ND	[37]
Italy	Raw and RTE vegetables, and frozen berries	ND	2012–2017	2.9% GI (*n* = 51)	ND	[39]
**OTHER COUNTRIES**						
Brazil	Fresh lettuce Green onions Strawberries	ND	2015–2016	0% (*n* = 12) 0% (*n* = 12) 0% (*n* = 12)	ND	[60]
Canada	RTE leafy greens	ND	2009	54% (*n* = 275)	Median: 5.0 × 10^2^ Range: 1.4–9 × 10^6^	[41]
Canada	Leafy greens	Belgium, Canada, France, US, Mexico, Spain, Poland/Serbia	2009–2010	28.2% (*n* = 641)	ND	[40]
Egypt	Green onion, watercress, radish, leek and lettuce	Egypt	2008–2009	20.8–34% (GI; *n* = 144) 0% (GII; *n* = 144)	Range: 4.2 × 10^3^–1.6 × 10^4^	[61]

^a^ Viral load is expressed as genome copies per 25 g of sample; RTE: ready-to-eat; ND: Not determined.

**Table 3:** Reductions of norovirus (NoV) and murine noroviurs (MNV) surrogate on food products by washing procedures that could be performed and/or transferred to private households provided that sanitizers were commercially available for consumers.

Food Product	Treatment	Log_10_ Reduction	Observations	Ref.
**Measurement of NoV Genomes**				
Blueberries, strawberries, raspberries, parsley	Stirring 15 g of food in 200 mL of tap water for 30 s + rinsing in 200 mL of tap water	0.1–1.5	Reductions on raspberries and parsley were only 0.1–0.9 log_10_	[68]
	Stirring 15 g of food in 200 mL of tap water containing 200 ppm FC for 30 s + rinsing in 200 mL of tap water	0–3.4	Reductions on raspberries and parsley were only 0–1.8 log_10_	[68]
Iceberg lettuce and perilla (mint) leaf	Immersion 5 g of food in 900 mL of water for 2 min + 30 s of rinsing water	0.9–1.3	Addition of a commercial class I detergent that can be used to wash fruits and vegetables made no difference	[69]
**Measurement of MNV infectivity**				
Iceberg lettuce	Shaking 50 g of food in 500 mL of tap water for 5 min + spin drying for 1 min	1.1	Considerable numbers of viruses were found in residual wash water	[70]
	Shaking 50 g of food in 500 mL of tap water containing 200 mg/L of sodium hypochlorite for 5 min + spin drying for 1 min	1.6–2.2	Effectiveness was greatly influenced by the presence of organic material	[70]
	Shaking 50 g of food in 500 mL of tap water containing 80 mg/L of PAA for 5 min + spin drying for 1 min	0.8		[70]
	Shaking 50 g of food in 500 mL of tap water containing 200 mg/L of PAA for 5 min + spin drying for 1 min	2.2		[70]
Romaine lettuce, blueberries, strawberries	Immersion of small portions of food in 10–15 mL solution containing 50 ppm FC for 1 min	0–2.5	Reductions differed by food (lettuce and strawberries < blueberries) The addition of Feclone™ significantly improved efficacy by >2 log_10_	[71]
	Immersion of small portions of food in 10–15 mL solution containing 85 ppm PAA for 1 min	2.5–3.5	The addition of Feclone™ improved efficacy by >2 log_10_ only in lettuce	[71]
	Immersion of small portions of food in 10–15 mL solution containing 20 ppm chlorine dioxide for 1 min	<1		[71]
Strawberries	Manual stirring of 25 g of food in 200 mL of water for 2 min + spray rinsing in 200 mL of potable water	1		[65]
	Manual stirring of 25 g of food in 200 mL of water containing sodium hypochlorite 50 ppm for 2 min + spray rinsing in 200 mL of potable water	1.5	Reductions observed for HAV and MS2 bacteriophage were 0.6–1.9 log_10_ higher	[65]
	Manual stirring of 25 g of food in 200 mL of a solution containing 0.5% LVA plus 0.5% SDS solution for 2 min + spray rinsing in 200 mL of potable water	1.4	Concentration of 5% LVA plus 2% SDS showed no significantly higher reductions	[65]
Blueberries and mixed berries	Soaking 10–20 g of food in 100 mL of distilled water for 1 min	1.6–1.8	As opposed to previous studies, spiking of berries was performed by immersion in a virus-containing solution, without a drying step	[66]
	Soaking 10–20 g of food in 100 mL of distilled water containing 100 ppm of FC for 1 min	3.8–4.2		[66]
	Soaking 10–20 g of food in 100 mL of distilled water containing 1% heat-denatured lysozyme for 1 min	4.1–4.2		[66]

NoV: Norovirus; FC: free chlorine; MNV: Murine Norovirus; PAA: peracetic acid; LVA: levulinic acid; SDS: sodium dodecyl sulphate; HAV: hepatitis A virus.

**Table 4:** Reported D-values (minutes required to achieve a log reduction at the indicated temperature) and z-values (number of degrees the temperature has to be increased to achieve a 10-fold reduction in the D-value) for the thermal inactivation of norovirus (NoV) and hepatitis A virus (HAV) in mussles.

	NoV GII (RTqPCR)	HAV (RTqPCR)	HAV (Infectious)	Ref.
D-value 60 °C (min)	25	NA	3.25–7.93	[76,77,78,79]
D-value 80 °C (min)	4.84	NA	3.2	[76,78]
D-value 90 °C (min)	NA	NA	0.55–0.9	[15]
D-value 100 °C (min)	0.93–1.3	1.58	0.38–0.86	[76,79,80]
z-value (°C)	28	NA	12.97–27.5	[15,76,77]

NA: Not available.
